# Genome-Wide Identification and Expression Analysis of Nitrate Transporter (NRT) Gene Family in *Eucalyptus grandis*

**DOI:** 10.3390/genes15070930

**Published:** 2024-07-17

**Authors:** Guangyou Li, Deming Yang, Yang Hu, Jianmin Xu, Zhaohua Lu

**Affiliations:** 1 Key Laboratory of State Forestry and Grassland Administration on Tropical Forestry, Research Institute of Tropical Forestry, Chinese Academy of Forestry, Guangzhou 510520, China; lgy@caf.ac.cn (G.L.); dmyang@caf.ac.cn (D.Y.); jianmxu@163.com (J.X.); 2 Xinhui Research Institute of Forestry Science, Jiangmen 529100, China; huyangchong@126.com

**Keywords:** *Eucalyptus grandis*, nitrogen, *EgNRTs*, RNA-Seq, expression profile, nitrate deficiency

## Abstract

*Eucalyptus grandis* is an important planted hardwood tree worldwide with fast growth and good wood performance. The nitrate transporter (NRT) gene family is a major core involved in nitrogen (N) absorption and utilization in plants, but the comprehensive characterization of *NRT* genes in *E. grandis* remains mostly elusive. In this study, a total of 75 *EgNRT* genes were identified from the genome of *E. grandis* that were distributed unevenly across ten chromosomes, except Chr9. A phylogenetic analysis showed that the EgNRT proteins could be divided into three classes, namely NRT1, NRT2 and NRT3, which contained 69, 4 and 2 members, respectively. The *cis*-regulatory elements in the promoter regions of *EgNRT* genes were mainly involved in phytohormone and stress response. The transcriptome analysis indicated that the differentially expressed genes of leaf and root in *E. grandis* under different N supply conditions were mainly involved in the metabolic process and plant hormone signal transduction. In addition, the transcriptome-based and RT-qPCR analysis revealed that the expression of 13 *EgNRT* genes, especially *EgNRT1.3*, *EgNRT1.38*, *EgNRT1.39* and *EgNRT1.52*, was significantly upregulated in the root under low-N-supply treatment, suggesting that those genes might play a critical role in root response to nitrate deficiency. Taken together, these results would provide valuable information for characterizing the roles of EgNRTs and facilitate the clarification of the molecular mechanism underlying *EgNRT*-mediated N absorption and distribution in *E. grandis*.

## 1. Introduction

Nitrogen (N) is an essential nutrient for plant growth and development, as it is required for the synthesis of chlorophyll, protein, nucleic acid and other secondary metabolites [1,2]. Under insufficient N supply, plant growth is inhibited, resulting in a decrease in product yield and quality. However, the excessive application of N fertilizer not only could not effectively increase yield but could also cause soil ecological damage and environmental pollution. The excessive use of N fertilizer leads to a serious waste of agricultural inputs in the long term. Therefore, understanding the biological and molecular mechanisms of N uptake and distribution in plants is critical to improve N-use efficiency and reduce nitrogen apply in crops.

Nitrate (NO_3_^−^) is one of the major forms of N uptake and utilization in higher plants [3]. Plants have developed high-affinity (HATS) and low-affinity nitrate-uptake systems (LATS) to cope with environments with low or high NO_3_^−^ concentrations to ensure an efficient N uptake [4]. Nitrate transporters (NRTs) have been proven to play a vital role in the nitrogen absorption of plants. The *NRT* family genes have been identified in many plants, such as cucumber [5], radish [6], rice [7], maize [8], *Populus* [9,10] and pineapple [11]. The *NRT* gene family could be classified into NRT1, NRT2 and NRT3. NRT1 belongs to the PEPTIDE TRANSPORTERS (PTR) family of the MAJOR FACILITATOR SUPER (MFS) family. AtNRT1.1 is the first identified NO_3_^−^ transporter that contributes to both low- and high-affinity N uptake. The expression of AtNRT1.1 is regulated by auxin signaling in both the shoots and roots [12], and AtNRT1.1 is involved in stomatal opening and drought susceptibility in *Arabidopsis* [13].

NRT2 belongs to the NITRATE/NITRITE PORTER (NNP) subfamily of MFS family. A total of seven *NRT2* genes have been identified in *Arabidopsis*, among which AtNRT2.1, AtNRT2.2, AtNRT2.4 and AtNRT2.5 are expressed in the root and participate in HATS. NRT2.1 and NRT2.2 are the major and minor contributors to the nitrate-inducible high-affinity transport system (IHATS), respectively. The disruption of NRT2.1 and NRT2.2 in *Arabidopsis* showed significant inhibition of HATS, and the N influx via the LATS was unaffected. The contribution of NRT2.2 was increased when the function of NRT2.1 was lost, leading to a partial compensation [14]. NRT2.4 is a nitrate transporter localized to the plasma membrane, which has a role in both the roots and shoots of *Arabidopsis* under N starvation [15]. NRT3, also called NAR2, is essential for HATS of the NRT2 protein in plants [16]. The interaction between NRT2 and NRT3 is required for HATS [17]. AtNRT3.1 interacts with almost all AtNRT2 family members except for AtNRT2.7 in *Arabidopsis* [18]. OsNAR2.1 interacted with OsNRT2.1, OsNRT2.2 and OsNRT2.3a to enhance their functions in NO_3_^−^ absorption and transport, but it cannot do so with OsNRT2.3b [19].

*E. grandis* is the widely planted hardwood tree worldwide [20]. Fast growth and good wood performance are the characteristics of *E. grandis*, which requires a large amount of nutrients, including N fertilizer. Therefore, the clarification of the molecular mechanisms underlying N uptake and utilization is crucial for NUE improvement in *E. grandis*. *NRT* genes, as the major nitrate transporter, and their function have been extensively characterized in several plants. The chromosomal-level genome of *E. grandis* was published [21], but the information on *EgNRT* genes was poorly understood. In this study, a total of 75 NRT genes were identified based on the genome of *E. grandis*. The chromosomal location, genetic structure and phylogenetic relationship of the *EgNRT* gene family were explored, and the expression profiles of *EgNRT* genes under low and high N supply were investigated. These findings would provide a basis for further studies on the functions of *EgNRT* genes and facilitate the clarification of the molecular regulatory network of N uptake and transportation in *E. grandis*.

## 2. Materials and Methods

### 2.1. Plant Materials

The experiment was carried out at the greenhouse of the Research Institute of Tropical Forestry, Chinese Academy of Forestry (23°11′ N, 113°23′ E), in 2023. *E. grandis* (Qinglong) clones were used as the experiment material and obtained from tissue culture. Eighteen healthy and uniform seedlings (seedling height about 39 cm and ground diameter of 3.5 mm) were planted into polypropylene containers with coconut husk. Nine plants each were treated with high N (HN: 119 mg/L) and low N (LN: 29.25 mg/L). The concentrations of other nutrient elements were similar for each treatment (P 15.5 mg/L, K: 298.0 mg/L, Mg: 48.1 mg/L, Ca: 210 mg/L, B: 0.5 mg/L, Mn: 0.5 mg/L, Zn: 0.5 mg/L, Cu: 0.5 mg/L, Mo: 0.5 mg/L and Fe: 5.6 mg/L). Each plant was watered with 100 mL of the corresponding nutrient solution. Each treatment contains 3 replicates and 3 plants for each replication. After the 24 h treatment, the leaves (on the third expanded leaf from the top) and roots for each treatment were sampled and stored in liquid nitrogen.

### 2.2. Identification and Characterization of NRT Genes in E. grandis

The sequences of 62 *Arabidopsis* NRT proteins were downloaded from the TAIR database (https://www.arabidopsis.org/, accessed on 5 April 2024). The public genome of *E. grandis* (GCA_000612305.2) was used as a reference genome for the custom database. To identify *EgNRT* genes, a BLASTP search according to AtNRT protein sequences was performed in *E. grandis* genomes with an E-value < 0.01. The potential sequences were submitted to the Pfam (http://pfam.sanger.ac.uk/search, accessed on 9 April 2024) and SMART (http://smart.embl-heidelberg.de/, accessed on 9 April 2024) to assess the NRT domains [22]. After removing the sequences without the PTR2, MFS1 or NAR2 domain, the reliable EgNRT proteins were obtained. The characterization of *EgNRT* genes, including the number of amino acids, the isoelectric point (pI), molecular weight (Mw) and grand average of hydropathicity (GRAVY), were calculated using the ProtParam tool of the ExPASy Server (https://web.expasy.org/protparam/, accessed on 11 April 2024).

### 2.3. Chromosomal Localization and Phylogenetic Analysis of EgNRT Proteins

The information about the chromosome localization of *EgNRT* genes was obtained from the GFF3 file of the *E. grandis* genome database and visualized using the TBtools software (v2.0906) according to the manufacturer’s instructions [23]. For the phylogenetic analysis, the multiple sequence alignments of AtNRT and EgNRT proteins were generated by Clustal W. Phylogenetic trees were constructed from multiple sequence alignments using the neighbor-joining method on the p-distance with pairwise deletion and 1000 bootstraKp replicates in MEGA 6. The phylogenetic tree was further visualized using Evolview (http://www.evolgenius.info/evolview/, accessed on 15 April 2024) [24].

### 2.4. Gene Structure and Conserved Motif Analysis of EgNRT Proteins

The conserved motifs of EgNRT protein were identified using multiple expectation maximization in the motif elicitation (MEME ver. 5.1.1, http://meme-suite.org, accessed on 15 April 2024) program, with a maximum of 10 motifs and a width of motifs of 6–50 aa. The information about gene structure for *EgNRT* genes was obtained from the GFF3 file of the *E. grandis* genome database. The gene structure and conserved motif within the phylogenetic tree of EgNRT proteins were visualized by TBtools software (v2.0906), according to the manufacturer’s instructions [23].

### 2.5. Cis-Element Analysis of EgNRT Gene Promoters

The upstream 2 kb sequence of the transcription start site (TSS) for each *EgNRT* gene was extracted by TBtools software as the presumptive promoter. The promoter sequences of *EgNRT* genes were loaded into the PlantCARE (http://bioinformatics.psb.ugent.be/webtools/plantcare/html/, accessed on 18 April 2024) database to identify potential *cis*-elements and further visualized by TBtools software (v2.0906) [23].

### 2.6. RNA Sequencing

The leaf and root tissues of *E. grandis* under low and high N supply were collected for transcriptome analysis. Total RNA was extracted by an RNA Simple Total RNA Extraction Kit (Tiangen biotech, Beijing Ltd. Co., Beijing, China), following the manufacturer’s instructions. The integrity, purity and concentration of total RNA were detected by 1% agarose gel electrophoresis and NanoDrop^®^ ND-1000 portable UV-Vis Spectrophotometer (Thermo Scientific, Waltham, MA, USA). RNA sequencing libraries were prepared as described previously [25]. Genes with FDR < 0.05 and |log2(foldchange)| ≥ 1 found by DESeq2 were assigned as differentially expressed. A gene ontology (GO) enrichment analysis of the differentially expressed genes (DEGs) was implemented by the GOseq R packages-based Wallenius non-central hyper-geometric distribution. KOBAS (v 3.0.3) software was employed to test the statistical enrichment of differential expression genes in the KEGG pathways. Heatmaps for expression profiles of EgNRT genes based on the transcriptome data were generated with TBtools software [23].

### 2.7. Real Time Quantitative Polymerase Chain Reaction (RT-qPCR)

Using a HiScript III 1st Strand cDNA Synthesis Kit (+gDNA wiper) (Vazyme Biotechnology Co., Nanjing, China), reverse transcripts qualified RNA samples into cDNA, following the manufacturer’s instructions. A RT-qPCR analysis was performed in a BioRad CFX96 Real-Time PCR platform (BioRad, Hercules, CA, USA). The specific program parameters were set according to the manufacturer’s instructions of Taq Pro Universal SYBR qPCR Master Mix (Vazyme Biotechnology Co., Nanjing, China). Three biological replicates were carried out. The relative expression levels were calculated by the 2^−ΔΔCT^ method, with the *Eucons08* gene as the internal references [26]. Primers for RT-qPCR were designed using NCBI Primer-BLAST software (v 2.15.0) and are listed in Supplementary Table S1.

### 2.8. Statistical Analysis

Data were analyzed using SPSS statistical software (SPSS 20.0, IBM, Armonk, NY, USA). One-way analysis of variance was used to determine statistical significance among multiple range tests.

## 3. Results

### 3.1. Genome-Wide Characterization of NRT Genes in E. grandis

To identify the candidate *EgNRT* genes, the sequences of 62 Arabidopsis NRT proteins were used to search through BLAST based on the *E. grandis* genome database. Subsequently, the retrieved putative genes were filtered through SMART and NCBI Batch CD search to remove proteins with incomplete domains. A total of 75 *EgNRT* family genes were obtained, including 69 EgNRT1/NPF, 4 EgNRT2 and 2 EgNRT3/NAR2 members (Table 1). The length of EgNRT proteins ranges from 205 aa (EgNRT3.1) to 649 aa (EgNRT1.29), and the molecular weight ranges from 22.29 (EgNRT3.1) to 71.60 (EgNRT1.29) kDa. In addition, the predicted pI values of the EgNRT proteins were in the range of 5.09 (EgNRT1.42)~9.66 (EgNRT1.2), and 61 EgNRT members belong to alkaline proteins (pI > 7). The instability index of EgNRT proteins ranges from 24.58 (EgNRT1.22) to 46.36 (EgNRT1.51), and six of the structures were unstable (instability index > 40). There were only three hydrophilic proteins (EgNRT1.11, EgNRT3.1 and EgNRT3.2) in EgNRT members, and the rest were hydrophobic proteins.

**Figure 1:**
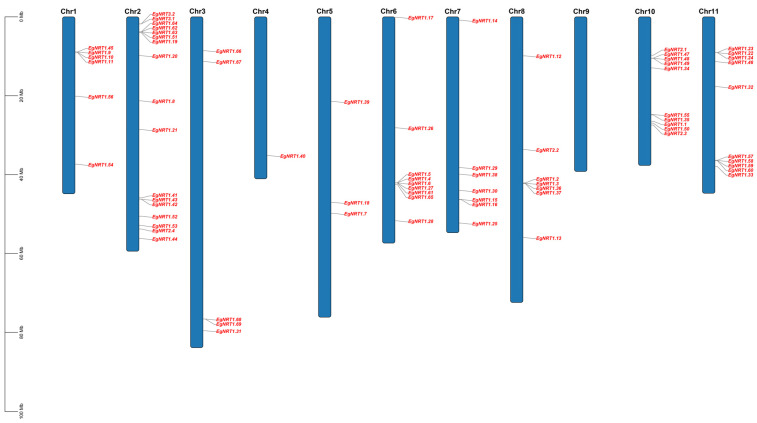
Chromosomal locations for the *EgNRT* genes on ten chromosomes. The chromosome number is represented at each bar top. Mb, megabase.

The chromosomal location analysis showed that 75 *EgNRT* family genes were distributed unevenly on the ten chromosomes (Chr) (Figure 1). The largest number of *EgNRT* genes was detected on Chr 2 (17 genes), followed by Chr 10 and 11, each with 10 genes. Chr6 possesses nine genes; two chromosomes (Chr7 and Chr8) contain seven genes; Chr1 contains six genes; and Chr3 and Chr5 have five and three genes, respectively. The lowest number was on Chr 4, with only one *EgNRT* gene. Moreover, no *EgNRT* member was identified on Chr 9. In addition, it was observed that many EgNRT1 members were located on Chr1, 2, 6, 8, 10 and 11 in adjacent positions, indicating a replication event during evolution.

### 3.2. Phylogenetic and Structure Analysis of the EgNRT Genes

**Figure 2:**
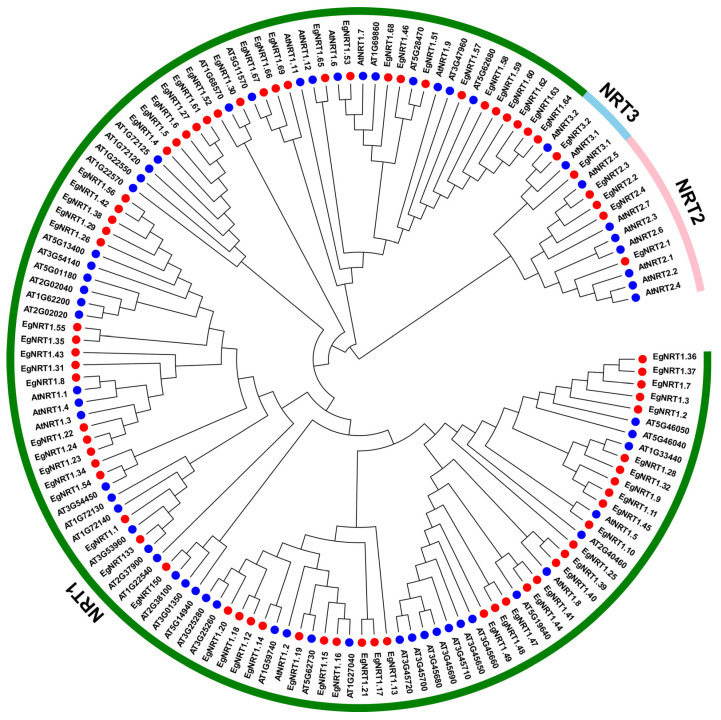
Phylogenetic analysis of NRT protein involved 62 Arabidopsis NRT protein and 75 *E. grandis* NRT protein sequences. Blue and red circles represent the AtNRT proteins and radish EgNRT proteins, respectively. The NRT1, NRT2 and NRT3 groups are marked in green, pink and sky blue, respectively.

**Figure 3:**
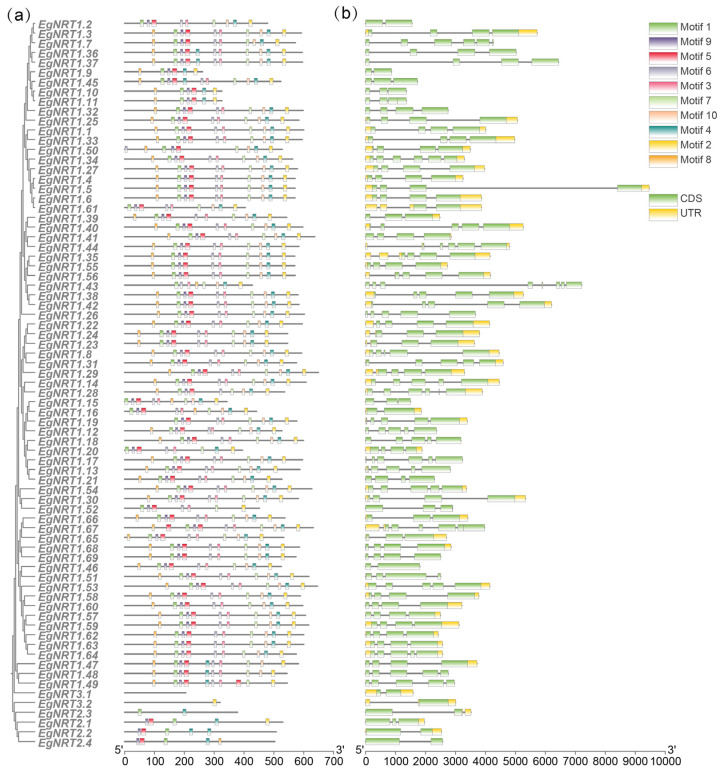
Phylogenetic analysis, motif distribution and gene structure of EgNRT genes. (**a**) Phylogenetic tree of 75 EgNRT proteins using the neighbor-joining method, and the conserved domain architecture of the EgNRT proteins. The motifs 1 to 10 are highlighted with different colored boxes. (**b**) Exon–intron structure for each *EgNRT* gene. The yellow bars indicate UTR, and the green bars refer to exons (CDS).

To compare the evolutionary relationships of the NRT gene family in *E. grandis*, a phylogenetic tree was generated using EgNRT and AtNRT full-length protein sequences (Figure 2). The result showed that the NRT protein of Arabidopsis and *E. grandis* could be divided into three classes—NRT1, NRT2 and NRT3—which contained 69, 4 and 2 EgNRT family members, respectively. The NRT1 class could be further subdivided into four groups. To investigate the structural characteristics of EgNRT proteins, ten conserved motifs were identified through a MEME database search (Figure 3a). The results show that most NRT1 members have 6~10 conserved motifs, and all of them contain motif 1, motif 5, motif 8 and motif 9, suggesting that those might be the most conserved motifs in the NRT1 family. However, the *NRT2* and *NRT3* genes possess 1~5 motif, and no conserved motif was identified in *EgNRT3.1*.

The gene structure of the EgNRT family was visualized to further explore the structural divergence (Figure 3b). The results showed that the length of *EgNRT* family gene sequences varied greatly, with the longest (*EgNRT1.5*) DNA sequence length approaching 10 kb, and the shortest (*EgNRT1.9*) less than 1 kb. The number distribution of the exon–intron structure of the *EgNRT* gene family members also varied greatly, with the exon number ranging from 2 to 8 and the intron number ranging from 1 to 7, among which *EgNRT1.43* has the highest exon number and eight EgNRT members have the lowest exon number. Many genes from the same branch are similar in structure. *EgNRT1.22*, *EgNRT1.23* and *EgNRT1.24* possess a similar structure and motif, suggesting that those genes might be functionally redundant. It is noteworthy that many genes from the same branch were different in structure, such as *EgNRT1.41/EgNRT1.44*, indicating that these genes might diverge functionally during evolution.

### 3.3. Cis-Element Analysis of EgNRT Genes Promoters

**Figure 4:**
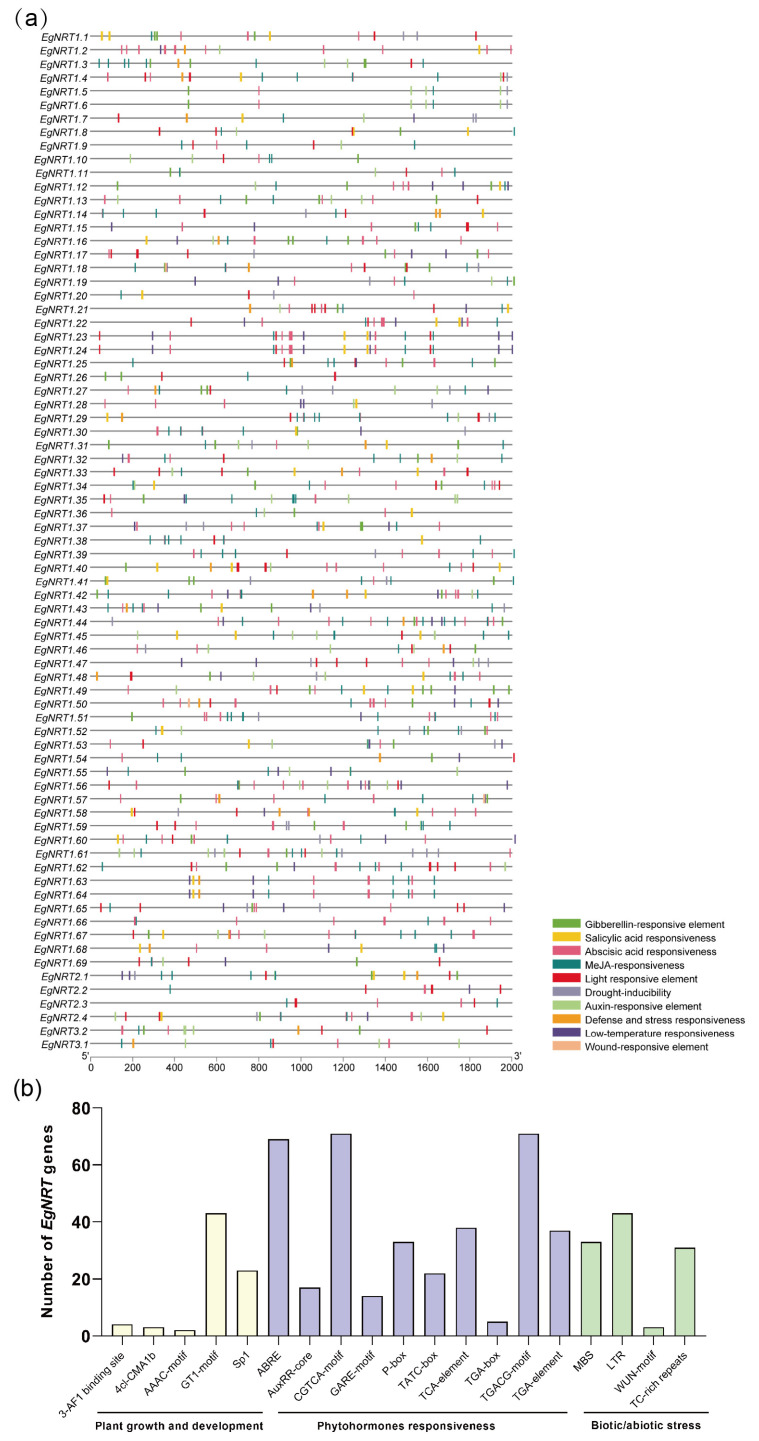
Characterization of *cis*-elements in the promoter regions of *EgNRT* genes. (**a**) Distribution of *cis*-elements in different colored rectangles. (**b**) The number of *EgNRT* genes harboring different *cis*-elements.

The characterization of cis-elements in the 2 kb upstream promoter sequences of *EgNRT* genes was analyzed by the PlantCARE database. Several crucial cis-elements involved in plant growth and development, phytohormones responsiveness and stress response were identified in the promoter region of *EgNRT* genes (Figure 4a and Supplementary Table S2). Approximately 71.2% of *EgNRT* genes contained light-responsive elements, including the 3-AF1 binding site, 4cl-CMA1b, 4cl-CMA2b, AAAC-motif, GT1-motif and Sp1, indicating that the expression of those *EgNRT* genes might be regulated by light signaling. Three auxin-responsive elements were found in the promoter region of 45 *EgNRT* genes, and the ABREs (abscisic acid-response elements) were distributed in 69 *EgNRT* genes. In addition, both the CGTCA motif and TGACG motif were identified in almost all *EgNRT* genes, suggesting that most *EgNRT* genes might participate in MeJA-mediated biological process. Gibberellin-responsive elements (GARE-motif, P-box and TATC-box) and a salicylic acid-responsive element (TCA-element) were found in the promoter region of 52 and 38 *EgNRT* genes. Moreover, about 45.2%, 58.9% and 42.3% of EgNRT family members contained drought-induction elements (MBS), low-temperature elements (LTR) and defense and stress responsiveness (TC-rich repeats), respectively (Figure 4b). The WUN-motif (wound-responsive element) exists in only three *EgNRT* genes (*EgNRT1.18*, *EgNRT1.50* and *EgNRT1.57*). These results indicate that the *EgNRT* genes were widely involved in plant growth and development, phytohormones responses and the stress-response process.

### 3.4. RNA-Seq Analysis of Root and Leaf in E. grandis under Low and High N Supply

**Figure 5:**
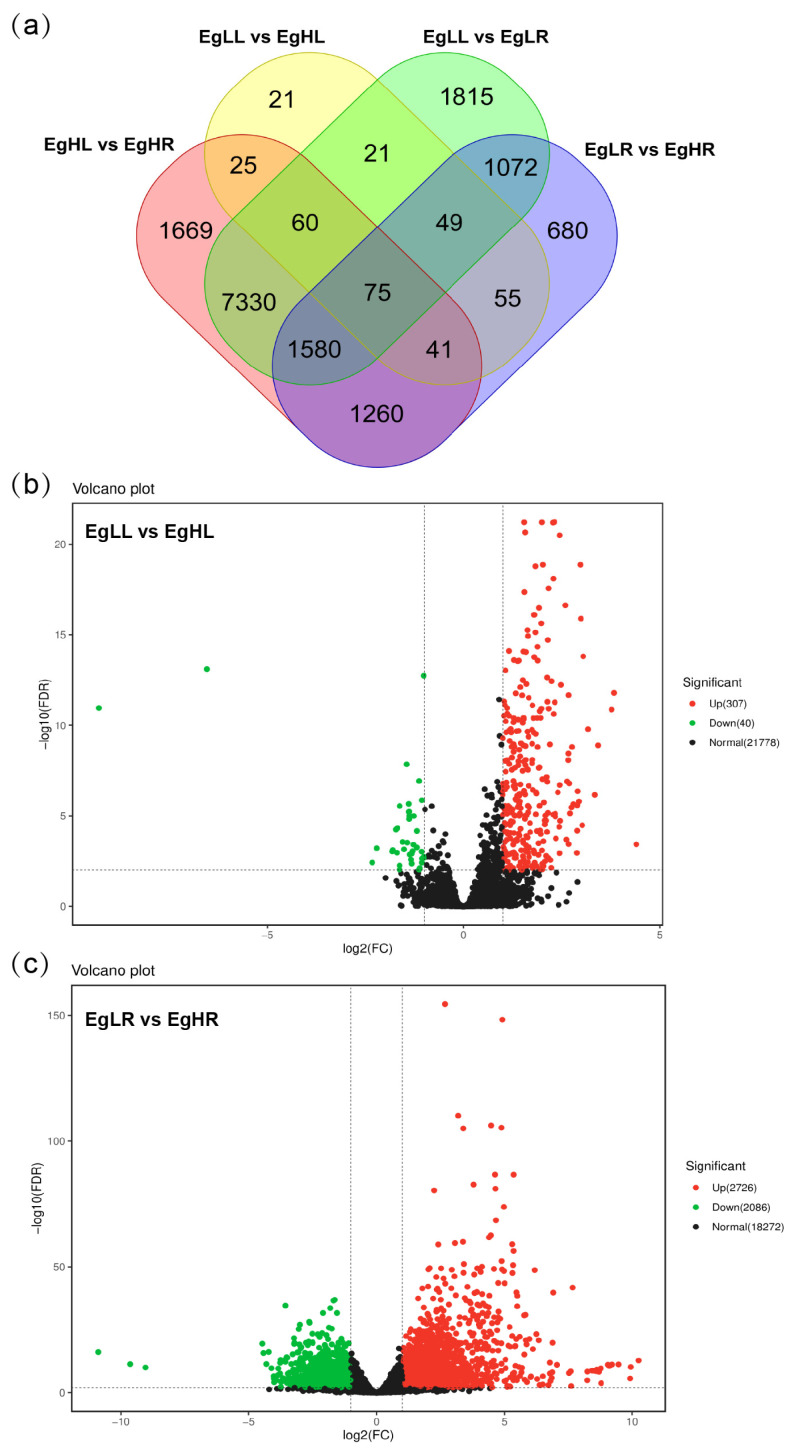
Differentially expressed genes (DEGs) of leaf and root in *E. grandis* under low- and high-N-supply conditions. (**a**) The display of DEGs in four groups. EgLL (leaf under low N supply), EgLR (root under low N supply), EgHL (leaf under high N supply) and EgHR (root under high N supply). (**b**) The upregulated and downregulated DEGs in EgLL vs. EgHL group. (**c**) The upregulated and downregulated DEGs in EgLR vs. EgHR group.

To investigate the nitrogen-response genes in Eucalyptus, an RNA sequencing (RNA-seq) analysis was performed with the leaf and root under low and high N supply. A total of 347 differentially expressed genes (DEGs, |Log2FoldChange| > 2, FDR < 0.05) were identified in the EgLL vs. EgHL group, including 307 upregulated and 40 downregulated DEGs (Figure 5a,b). For the EgLR vs. EgHR group, a total of 4812 DEGs were identified, including 2726 upregulated and 2086 downregulated DEGs (Figure 5c). The gene ontology (GO) term enrichment analysis showed that the DEGs of the EgLL vs. EgHL and EgLR vs. EgHR group were mainly involved in metabolic process, cellular process, membrane, membrane part, binding and catalytic process (Supplementary Figure S1). The Kyoto Encyclopedia of Genes and Genomes (KEGG) enrichment analysis showed that the DEGs of the EgLL vs. EgHL group were mainly involved in plant hormone signal transduction, amino sugar and nucleotide sugar metabolism, and plant–pathogen interaction, while the DEGs of the EgLR vs. EgHR group were responsible for plant hormone signal transduction, ribosomes, phenylpropanoid biosynthesis and carbon metabolism (Supplementary Figure S2).

### 3.5. Expression Profiles of EgNRT Genes under Low and High N Supply

**Figure 6:**
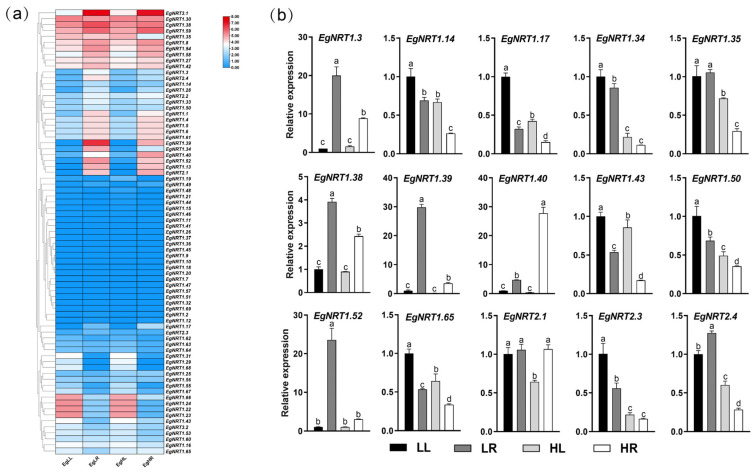
The expression profiles of *EgNRT* genes in leaf and root under different N-supply conditions. (**a**) Heatmap showing *EgNRT* genes expression pattern in leaf and root under different N-supply conditions. (**b**) Validation of 15 *EgNRT* genes’ expression data from RNA-Seq by RT-qPCR. Different lowercase letters indicate significant differences at level *p*-value = 0.05.

The expression profiles of *EgNRT* genes were further analyzed using the transcriptome data (Figure 6a). As expected, tissue-specific and N-response expressions were observed for *EgNRT* genes. Among all *EgNRT* genes, 24 members showed no expression (FPKM < 1) in the four samples. *EgNRT1.13*, *EgNRT1.17*, *EgNRT1.34*, *EgNRT1.39*, *EgNRT1.40*, *EgNRT1.52* and *EgNRT2.1* were mainly expressed in the leaf, while *EgNRT1.22*, *EgNRT1.23*, *EgNRT1.24*, *EgNRT1.29*, *EgNRT1.31*, *EgNRT1.55*, *EgNRT1.66*, *EgNRT1.67*, *EgNRT1.68* and *EgNRT3.1* were mainly expressed in the root. Compared to the low-N treatment, the expressions of four *EgNRT* genes, namely *EgNRT1.19*, *EgNRT1.25*, *EgNRT1.29* and *EgNRT3.1*, were significantly induced in the leaf under high-N conditions. In addition, the expression of *EgNRT1.3*, *EgNRT1.14*, *EgNRT1.34*, *EgNRT1.35*, *EgNRT1.38*, *EgNRT1.39* and *EgNRT2.3* had little changes in the leaf but was significantly downregulated in the root under high-N conditions. On the contrary, the expressions of *EgNRT1.17*, *EgNRT1.40*, *EgNRT1.43*, *EgNRT1.65* and *EgNRT2.1* were significantly downregulated in the root. It is noteworthy that the expression of *EgNRT2.4* was upregulated in the leaf and downregulated in the root. To verify the reliability of transcriptome data, the expression patterns of 15 *EgNRT* genes were evaluated by RT-qPCR analysis. The results revealed that the expression trends of these genes were basically in accordance with the transcript-abundance changes from RNA-seq data (Figure 6b). Compared to low-N treatment, the expressions of *EgNRT1.14*, *EgNRT1.17*, *EgNRT1.34*, *EgNRT1.35*, *EgNRT1.43*, *EgNRT1.50*, *EgNRT1.65*, *EgNRT2.3* and *EgNRT2.4* were significantly downregulated in both the leaf and root. The expressions of *EgNRT1.3*, *EgNRT1.38*, *EgNRT1.39* and *EgNRT1.52* were significantly downregulated in the root, indicating that those genes might play vital roles in the root’s NO_3_^−^ transport process. In addition, *EgNRT1.40* was significantly upregulated in the root, suggesting that it might be involved in LATS. Moreover, the expression of *EgNRT2.1* was significantly downregulated in the leaf, demonstrating that it might participate in the N distribution of the leaf under high N supply.

## 4. Discussion

*NRT* genes play vital roles in regulating plant N uptake and distribution to adapt to different nutrient conditions. The *NRT* family genes have been identified in several herbs and wood plants, such as cucumber [5], radish [6], rice [7], maize [8], *Populus* [9,10] and pineapple [11]. Up until now, the identification and characterization of the *EgNRT* gene family have not been comprehensively investigated, thus creating a barrier to the genetic improvement of NUE in *E. grandis* plants. Here, a total of 75 *EgNRT* genes were identified at the genome level based on the *E. grandis* genome. The isoelectric point (pI), molecular weight, chromosome location, genetic structure, domain architecture, phylogenetic relationship and expression profiles of these *EgNRT* genes were characterized (Table 1); this was the first systematic characterization of the NRT gene family in *E. grandis*. Similar to other plants, the EgNRT gene family contains a large number of NRT1 and a few NRT2 and NRT3 gene members [5,7,10]. Compared with *NRT* genes in *Arabidopsis*, the number of *EgNRT* genes have only a slight increase. Several *NRT* genes were highly conserved in *Arabidopsis* and *E. grandis* during evolution, indicating that the functions of these genes are relatively well conserved.

Besides being a nutrient, nitrate also plays an important signaling role in plant development and environmental response; the interactions between the signaling pathways of nitrate and phytohormones to bring about changes in physiology and morphology have been revealed [12,27,28,29]. Nitrate could regulate the biosynthesis and signaling of phytohormones, including auxin, cytokinin, GA, ABA, MeJA and SA, while the feedback from hormonal signaling modulates the absorption and metabolism of NO_3_^−^ [15,30,31]. In this study, many cis-elements involved in the phytohormonal response were found in the promoters of *EgNRT* genes (Figure 4a and Supplementary Table S1), indicating that *EgNRT* genes were integrated into the growth regulatory network mediated by phytohormones. Furthermore, the DEGs of root and leaf in *E. grandis* under different N condition were mainly related to the plant hormone signal transduction pathway based on the KEGG-enrichment analysis (Supplementary Figure S2), indicating that similar interactions between the signaling pathways of nitrate and phytohormones might exist in *E. grandis*. Furthermore, the exploration of the transcriptional regulatory network of nitrogen uptake and distribution mediated by *EgNRT* genes can facilitate our understanding of the interaction between nitrate and phytohormones’ signaling.

The NRT family members perform diverse functions in the uptake and distribution of nitrate across the entire plant organism [3]. The tissue-specific expression of NRT-family genes has been observed in several species, such as Arabidopsis, rice, cucumber and poplar [5,9,16,32]. Herein, the transcriptome and RT-qPCR analysis showed that most of the *EgNRT* genes exhibited leaf- or root-specific expression patterns, suggesting that *EgNRT* genes were involved in regulating the development of different tissues (Figure 6). *EgNRT1.13*, *EgNRT1.17*, *EgNRT1.34*, *EgNRT1.39*, *EgNRT1.40*, *EgNRT1.52* and *EgNRT2.1* have higher expression in the leaf, while in the root, it was undetected, suggesting that its role in leaf rather than root, while *EgNRT1.22*, *EgNRT1.23*, *EgNRT1.24*, *EgNRT1.29*, *EgNRT1.31*, *EgNRT1.55*, *EgNRT1.66*, *EgNRT1.67*, *EgNRT1.68* and *EgNRT3.1* were the opposite. AtNRT1.1 was the first identified and the most extensively explored NRT member in *Arabidopsis*, and it is expressed widely in roots and shoots [33,34,35]. As a homologous gene, *EgNRT1.8* displayed a higher expression in the root. The expression levels of most *EgNRT* genes were upregulated at a NO_3_^−^ concentration, which may lead to the strong nitrogen absorption ability under low-N-supply conditions in *E. grandis*. The subfamily NRT2 belongs to the high-affinity transport system proteins, which play critical roles in N uptake at low nitrate concentrations [36,37]. Among the four EgNRT2 members identified in this study, the expressions of *EgNRT2.3* and *EgNRT2.4* were induced by a low-N-supply condition in the leaf and root (Figure 6), indicating that *EgNRT2.3* and *EgNRT2.4* play an essential role in nitrate acquisition and remobilization. It is worth noting that the expression of *EgNRT2.1* was significantly downregulated only in the leaf. *EgNRT2.2* showed a stable expression trend under different NO_3_^−^ concentrations, suggesting that it may be a constitutive sensor, particularly in the roots, which are the primary uptake sites for nitrate. The precise exact function of these *EgNRT* genes in response to low-nitrogen conditions remains to be further investigated.

## Figures and Tables

**Table 1:** Characterization of EgNRT family genes in *E. grandis*.

Gene Name	Gene ID	Chromosome	Start	End	Length (aa)	Molecular Weight (kDa)	Theoretical pI	Instability Index	Gravy
EgNRT1.1	KAK3410094.1	Chr10	26,452,832	26,456,837	600	66.50	8.99	41.06	0.167
EgNRT1.2	KAK3417242.1	Chr8	42,098,203	42,099,747	479	53.77	9.66	34.99	0.258
EgNRT1.3	KAK3417244.1	Chr8	42,112,816	42,118,538	592	66.43	9.28	31.56	0.228
EgNRT1.4	KAK3426575.1	Chr6	42,080,525	42,083,773	571	62.29	6.90	39.33	0.410
EgNRT1.5	KAK3426576.1	Chr6	42,080,525	42,089,987	571	62.25	6.83	40.74	0.413
EgNRT1.6	KAK3426577.1	Chr6	42,086,125	42,089,987	571	61.94	6.83	40.73	0.424
EgNRT1.7	KAK3431236.1	Chr5	49,790,727	49,794,988	572	63.81	9.15	34.49	0.283
EgNRT1.8	KAK3441088.1	Chr2	21,269,805	21,274,259	593	65.43	9.03	35.09	0.285
EgNRT1.9	KAK3444550.1	Chr1	8,907,636	8,908,499	261	29.19	8.99	32.01	0.183
EgNRT1.10	KAK3444551.1	Chr1	8,912,563	8,913,917	326	36.52	8.21	37.83	0.082
EgNRT1.11	KAK3444552.1	Chr1	8,962,732	8,964,089	326	36.59	8.79	39.60	−0.012
EgNRT1.12	KAK3415186.1	Chr8	9,882,107	9,884,471	527	58.26	9.60	28.56	0.308
EgNRT1.13	KAK3418198.1	Chr8	55,988,616	55,991,440	587	65.54	8.96	37.33	0.271
EgNRT1.14	KAK3419514.1	Chr7	828,051	832,508	608	67.37	9.06	45.91	0.187
EgNRT1.15	KAK3421958.1	Chr7	46,265,830	46,267,324	343	37.87	8.94	45.76	0.372
EgNRT1.16	KAK3421969.1	Chr7	46,339,254	46,341,108	442	48.17	9.25	36.47	0.333
EgNRT1.17	KAK3423094.1	Chr6	77,443	80,673	596	65.65	9.11	31.77	0.292
EgNRT1.18	KAK3431109.1	Chr5	47,014,489	47,017,668	599	66.28	9.01	32.54	0.492
EgNRT1.19	KAK3440113.1	Chr2	4,017,890	4,021,277	577	63.27	9.12	42.37	0.407
EgNRT1.20	KAK3440606.1	Chr2	9,819,211	9,821,092	395	43.55	8.51	30.53	0.475
EgNRT1.21	KAK3441424.1	Chr2	28,576,333	28,578,626	527	58.26	8.38	36.16	0.433
EgNRT1.22	KAK3404455.1	Chr11	9,062,109	9,066,244	595	65.80	8.82	24.58	0.234
EgNRT1.23	KAK3404456.1	Chr11	9,062,616	9,066,244	546	60.81	8.90	25.43	0.243
EgNRT1.24	KAK3404457.1	Chr11	9,062,446	9,066,244	546	60.81	8.90	25.43	0.243
EgNRT1.25	KAK3422698.1	Chr7	52,269,257	52,274,320	584	65.32	8.99	34.99	0.254
EgNRT1.26	KAK3425311.1	Chr6	28,102,756	28,106,418	602	66.84	9.56	34.21	0.211
EgNRT1.27	KAK3426574.1	Chr6	42,076,124	42,080,091	579	62.82	6.03	37.39	0.336
EgNRT1.28	KAK3427971.1	Chr6	51,701,358	51,705,252	536	59.13	8.02	42.99	0.253
EgNRT1.29	KAK3421328.1	Chr7	38,221,725	38,225,022	649	71.60	9.02	36.52	0.252
EgNRT1.30	KAK3421701.1	Chr7	44,006,688	44,012,020	582	64.52	8.96	32.12	0.260
EgNRT1.31	KAK3439365.1	Chr3	79,521,298	79,525,885	576	63.02	9.04	31.88	0.358
EgNRT1.32	KAK3405187.1	Chr11	17,678,784	17,681,533	598	66.32	9.35	34.37	0.077
EgNRT1.33	KAK3406913.1	Chr11	37,923,342	37,928,314	595	66.16	9.01	39.35	0.274
EgNRT1.34	KAK3409078.1	Chr10	12,946,580	12,949,871	563	61.71	9.07	34.88	0.401
EgNRT1.35	KAK3409870.1	Chr10	24,836,710	24,840,859	570	63.65	8.69	24.92	0.198
EgNRT1.36	KAK3417246.1	Chr8	42,197,856	42,202,878	595	65.56	9.17	31.11	0.287
EgNRT1.37	KAK3417247.1	Chr8	42,217,236	42,223,672	596	65.61	9.26	33.40	0.286
EgNRT1.38	KAK3421394.1	Chr7	39,895,137	39,900,397	581	64.11	5.63	31.42	0.271
EgNRT1.39	KAK3430197.1	Chr5	21,446,821	21,449,295	543	60.23	5.78	32.58	0.170
EgNRT1.40	KAK3434719.1	Chr4	35,140,519	35,145,774	597	66.46	6.76	32.68	0.153
EgNRT1.41	KAK3442420.1	Chr2	45,896,146	45,898,996	637	70.20	6.05	29.04	0.303
EgNRT1.42	KAK3442480.1	Chr2	46,229,753	46,235,956	584	64.35	5.09	32.84	0.297
EgNRT1.43	KAK3442482.1	Chr2	46,237,521	46,244,731	428	47.59	6.15	38.10	0.212
EgNRT1.44	KAK3443531.1	Chr2	56,169,871	56,174,670	584	64.21	7.79	28.69	0.328
EgNRT1.45	KAK3444549.1	Chr1	8,900,093	8,901,821	523	58.40	8.37	39.65	0.375
EgNRT1.46	KAK3404606.1	Chr11	11,376,427	11,378,235	525	57.69	9.20	39.38	0.447
EgNRT1.47	KAK3408803.1	Chr10	10,440,073	10,443,789	582	63.44	8.90	41.94	0.360
EgNRT1.48	KAK3408804.1	Chr10	10,462,388	10,465,144	544	59.73	8.80	36.80	0.347
EgNRT1.49	KAK3408805.1	Chr10	10,487,412	10,490,375	545	59.40	9.09	35.42	0.287
EgNRT1.50	KAK3410139.1	Chr10	26,946,074	26,949,565	528	59.27	8.18	45.59	0.324
EgNRT1.51	KAK3440094.1	Chr2	3,808,628	3,811,132	617	68.95	9.40	46.36	0.183
EgNRT1.52	KAK3442757.1	Chr2	50,546,560	50,549,464	452	50.09	8.43	29.64	0.146
EgNRT1.53	KAK3443072.1	Chr2	52,890,529	52,894,671	646	70.57	9.21	38.27	0.277
EgNRT1.54	KAK3446521.1	Chr1	37,349,479	37,352,842	627	69.13	8.39	36.76	0.162
EgNRT1.55	KAK3409854.1	Chr10	24,732,599	24,735,325	570	63.96	8.85	28.49	0.249
EgNRT1.56	KAK3445120.1	Chr1	20,129,841	20,134,001	571	63.91	8.12	24.92	0.206
EgNRT1.57	KAK3406767.1	Chr11	36,353,120	36,355,616	606	67.99	9.11	38.27	0.182
EgNRT1.58	KAK3406769.1	Chr11	36,380,891	36,384,668	589	64.90	9.28	34.47	0.345
EgNRT1.59	KAK3406773.1	Chr11	36,422,757	36,425,873	617	68.93	8.59	35.14	0.176
EgNRT1.60	KAK3406774.1	Chr11	36,444,862	36,448,073	597	66.05	9.21	38.63	0.253
EgNRT1.61	KAK3426578.1	Chr6	42,086,125	42,089,987	404	44.60	6.74	39.95	0.460
EgNRT1.62	KAK3440091.1	Chr2	3,792,284	3,794,705	600	66.73	9.31	35.27	0.270
EgNRT1.63	KAK3440092.1	Chr2	3,808,628	3,811,132	600	66.84	9.28	34.88	0.251
EgNRT1.64	KAK3440093.1	Chr2	3,803,834	3,806,399	575	64.00	9.30	35.81	0.202
EgNRT1.65	KAK3426647.1	Chr6	42,459,508	42,462,203	533	58.96	8.86	43.17	0.205
EgNRT1.66	KAK3435815.1	Chr3	8,569,729	8,573,136	537	58.92	7.50	37.84	0.447
EgNRT1.67	KAK3435907.1	Chr3	11,297,439	11,301,408	632	69.37	5.98	41.14	0.402
EgNRT1.68	KAK3439164.1	Chr3	76,588,303	76,591,154	586	64.67	9.29	36.47	0.256
EgNRT1.69	KAK3439166.1	Chr3	76,595,223	76,597,726	574	63.13	9.11	43.35	0.360
EgNRT2.1	KAK3408717.1	Chr10	9,727,832	9,729,792	530	57.47	8.86	39.08	0.324
EgNRT2.2	KAK3416771.1	Chr8	33,614,452	33,616,983	508	54.83	8.51	35.42	0.390
EgNRT2.3	KAK3410216.1	Chr10	27,433,602	27,437,118	378	39.93	6.29	33.94	0.580
EgNRT2.4	KAK3443186.1	Chr2	53,783,851	53,786,418	503	54.02	8.82	34.37	0.407
EgNRT3.1	KAK3439874.1	Chr2	1,786,661	1,788,242	205	22.29	9.21	38.46	−0.092
EgNRT3.2	KAK3439873.1	Chr2	1,769,131	1,772,134	320	35.90	6.26	38.76	−0.238

## Data Availability

The data reported in this paper were deposited in the Genome Sequence Archive (Genomics, Proteomics and Bioinformatics 2021) in National Genomics Data Center, China National Center for Bioinformation/Beijing Institute of Genomics, Chinese Academy of Sciences (GSA: CRA017129), and are publicly accessible at https://ngdc.cncb.ac.cn/gsa (accessed on 17 June 2020).

## Supplementary Materials

The following supporting information can be downloaded at https://www.mdpi.com/article/10.3390/genes15070930/s1, Figure S1: GO analysis of DEGs in EgLL vs. EgHL and EgLR vs. EgHR group. BP, biological process. CC, cellular component. MF, molecular function; Figure S2: KEGG-enrichment analysis of DEGs in EgLL vs. EgHL and EgLR vs. EgHR group; Table S1: The potential cis-elements in the promoter region of 75 *EgNRT* genes; Table S2: Primers used in this study.
