# Supplementary figures and images for: Genome-Wide Identification and Expression Analysis of Nitrate Transporter (NRT) Gene Family in Eucalyptus grandis
Source: Genes (Basel). 2024 Jul 17;15(7):930. doi: 10.3390/genes15070930 (PMC11275818; doi:10.3390/genes15070930)

## EgLR vs EgHR

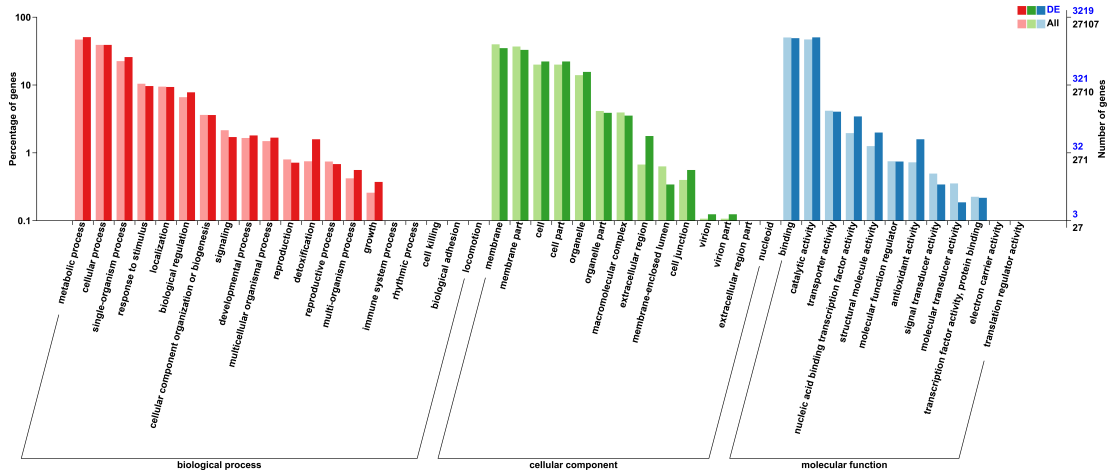

Supplement: Supplementary file 1 [file genes-15-00930-s001.zip › Fig.S1.pdf]

## EgLL vs EgHL

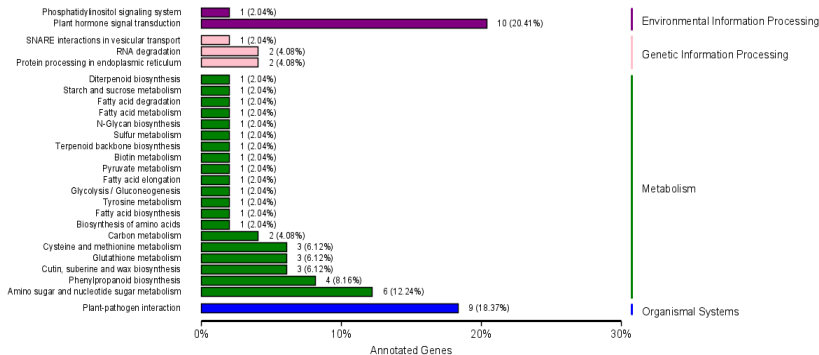

## EgLR vs EgHR

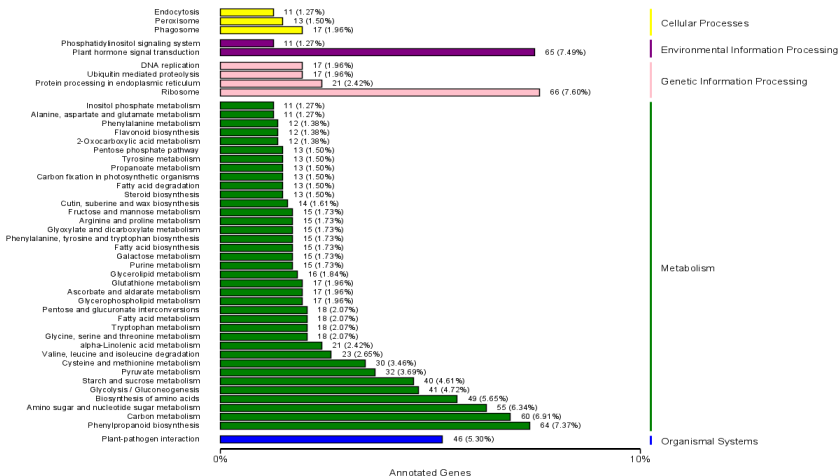

Supplement: Supplementary file 1 [file genes-15-00930-s001.zip › Fig.S2.pdf]
